# Solid-State Gas Sensors: Sensor System Challenges in the Civil Security Domain

**DOI:** 10.3390/ma9010065

**Published:** 2016-01-20

**Authors:** Gerhard Müller, Angelika Hackner, Sebastian Beer, Johann Göbel

**Affiliations:** 1 Department of Applied Sciences and Mechatronics, Munich University of Applied Sciences, Lothstraße 34, München D-80335, Germany; 2 Airbus Group Innovations, Munich D-81663, Germany; angelika.hackner@airbus.com (A.H.); sebastian.beer@infineon.com (S.B.); johann.goebel@airbus.com (J.G.); 3 Infineon Technologies, Wernerwerkstraße 2, Regensburg D-93049, Germany

**Keywords:** explosives, drugs, gas sensor, electrostatic precipitation, solid-vapor conversion, surface ionization

## Abstract

The detection of military high explosives and illicit drugs presents problems of paramount importance in the fields of counter terrorism and criminal investigation. Effectively dealing with such threats requires hand-portable, mobile and affordable instruments. The paper shows that solid-state gas sensors can contribute to the development of such instruments provided the sensors are incorporated into integrated sensor systems, which acquire the target substances in the form of particle residue from suspect objects and which process the collected residue through a sequence of particle sampling, solid-vapor conversion, vapor detection and signal treatment steps. Considering sensor systems with metal oxide gas sensors at the backend, it is demonstrated that significant gains in sensitivity, selectivity and speed of response can be attained when the threat substances are sampled in particle as opposed to vapor form.

## 1. Introduction

Suicide attacks and roadside bombs have become common threats to the civilian lives in many countries [1]. Frequent targets of attacks are human crowds and critical pieces of infrastructure. Important targets with a very high damage potential are also the entire fields of air and ground traffic. Adequate methods of counteracting such attacks are seen in restricting the access to potential targets and performing controls on persons and goods entering potential target sites. A second related problem with international dimensions is the trafficking of illicit drugs [2,3]. Similar to the abovementioned problems with improvised explosive devices, efficient countermeasures are seen in establishing easily-deployable and frequently-changing police control points along the trafficking routes.

Protecting the freedom and security of Europe and its citizens is one of the key objectives of the European Union (EU) and a major subject in the FP7 and Horizon 2020 programs [4,5]. Measures supported by the EU range from general and holistic approaches to chemical, biological, radioactive, nuclear and explosive (CBRNE) preparedness and resilience [6,7] up to the development of specific supporting technologies to ensure CBRNE security [8,9,10,11]. The arena of CBRNE sensor technologies, in particular, has proven to be a very attractive field to researchers with a background in gas sensors and gas sensor materials. These and related development programs in the U.S. [12], in fact, have produced a great deal of innovative and very promising sensor approaches [13], which however have not yet made a significant impact in the security sensor market. The tacit assumption in almost all of this innovative work is that the gas sensors in question need to be able to detect in a direct manner the extremely small concentrations of target gases that emerge from military high explosives and from illicit drugs. As the equilibrium vapor pressures of almost all of these materials are far below any of those concentrations of reactive gases that normally abound in the ambient air, the detection of security threats requires extremely sensitive and selective sensor devices. Extreme sensitivity and selectivity, in principle, can be attained by sampling huge amounts of air through pre-concentrators, which contain highly selective adsorbents [14,15]. The collected target molecules can then be flash-evaporated into less sensitive and less selective backend vapor detectors to generate an alarm. Even if such pre-concentrator detector solutions can be devised, the very long sampling times required usually do not conform to the tight time constraints that exist in typical security monitoring applications.

**Figure 1:**
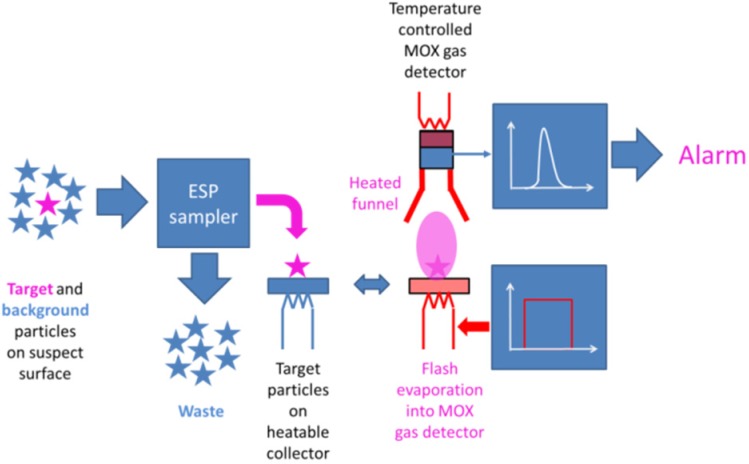
Sensor system for sampling, sorting and converting low vapor pressure target particles into detectable vapors. ESP, electrostatic precipitation.

The point we would like to make in this paper is that the tight sensitivity, selectivity and time constraints of typical security monitoring applications are much easier to meet if the gas sensors are embedded into integrated sensor systems, like the one shown in Figure 1. With such systems, the target substances are acquired in the form of solid particle residue, and the collected residue is flash-evaporated into a backend vapor detector.

In the following, we report on feasibility tests in which all of these single steps in the detection chain have been tested and evaluated in separation. As a first result, we show that metal oxide (MOX) gas sensors with a resistive (RES) or a surface ionization (SI) readout are able to detect explosives and illicit drugs with sensitivities sufficient to live up to security sensing requirements (Section 4.1 and Section 4.2). As a second result, we demonstrate in Section 4.3 that such sensors can be endowed with a remarkable degree of selectivity, once the particle residue is gathered from the environment with the help of innovative electrostatic particle samplers [16]. In such samplers, electrostatic precipitation (ESP) processes are used to deposit purified samples of either high electron or high proton affinity matter on hotplate heaters from where they can be evaporated into the backend MOX detectors. Overall, we want to demonstrate that particle collection is inherently fast, sensitive and, last, but not least, surprisingly selective.

Before we proceed to a discussion of our results, we provide some background concerning the target materials in question (Section 2) and also on ion mobility spectrometry (IMS) [17] (Section 3). The latter is done, firstly, because IMS technology is the benchmark technology against which every new development in the security sensor domain should be compared. Secondly, we consider IMS because the atmospheric pressure chemical ionization (APCI) processes operative in IMS instruments are also operative in the ESP samplers and because these endow our alternative sensor systems with a remarkable degree of selectivity, which would be hard to attain with the backend MOX detectors alone. In the concluding Section 5, we summarize and briefly consider alternative sensor and sensor system technologies, which we consider interesting for building improved security threat detectors in the future.

## 2. Target Substances

As already mentioned, a common problem in the field of security sensor research is that security sensing problems are misconceived as straight-forward gas sensing problems. In order to avoid such misconceptions, we show in the following two sections that explosives and illicit drugs normally abound in the form of low vapor pressure solids. As a consequence, both kinds of threat materials first need to be collected in the form of particle residue and then transformed into detectable vapors.

### 2.1. Explosives

The vast variety of potentially explosive materials can be loosely subdivided into the groups of military high explosives and improvised explosives. Typical members of both groups are the following:

- Military high explosives: trinitrotoluol (TNT), cyclotrimethylene-trinitramine (RDX), pentaerythritol tetranitrate (PETN);
- Improvised explosives: ammonium nitrate (AN), urea nitrate (UN).

**Figure 2:**
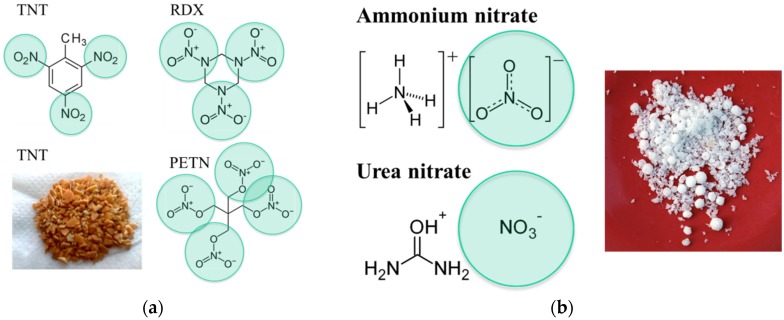
Molecular structure of explosives: (**a**) military high explosives; (**b**) improvised explosives. All substances contain strongly electron-attracting NO*_x_* side groups (marked by green circles.

A common property of all those materials, and an obstacle with regard to their detection, is that all of these abound in solid form at normal ambient temperature and pressure conditions (Figure 2) and that thus these are not normally detectable using common types of solid-state gas sensors. This latter fact can be easily seen by considering the vapor pressure data compiled in Table 1. The common reason for the low volatility of explosives is that these abound in the form of molecular solids with a high binding energy within the constituent molecules themselves and a low binding energy between nearest-neighbor molecules. In terms of macroscopic properties, this small inter-molecule binding energy translates into low melting and boiling points. Table 1 shows that melting points are in general well below 200 °C, while boiling points are in the order of 150–300 °C. Table 1 also shows that boiling often coincides with molecular decomposition. Once vaporized, the solids decompose into individual molecules, and these further decompose into smaller fragments. Experimentally, it was found that all explosives produce a cloud of reaction products, most notably nitrogen dioxide (NO_2_), nitric oxide (NO), ammonia (NH_3_), hydrogen (H_2_), carbon monoxide (CO), carbon dioxide (CO_2_), nitrogen (N_2_), oxygen (O_2_) and methane (CH_4_) [18]. Among those, NO_2_ is the one that is most easily detected at low concentrations and with relative selectivity using solid state gas sensors. The basic reason for its easy detectability is its exceptionally high electron affinity, which sets it aside from most other gases in the ambient air (Table 2) and which enables it to extract very efficiently conduction electrons from semiconductor materials.

**Table 1:** Molecular properties of important explosives materials. Most electron affinities are theoretical estimates [19].

Name	Comment	Aggregate Form	Electron Affinity (eV)	Equil. Vapor Pressure at 25 °C (ppb)	Water Solubility (g/L)	Melting Point (°C)	Boiling Point (°C)	Product Gas
**TNT [20]**	Military high explosive	Solid	~2.3	9.55	0.13 (20 °C)	80	295 (igniting)	NO_2_
**RDX [21]**	Military high explosive	Solid	~1.2	0.006	insoluble	205	235	NO_2_
**PETN [22]**	Military high explosive	Solid	~1.6	0.18	0.1 (50 °C)	141	150 (de-com-poses)	NO_2_
**Ammonium nitrate (AN) [23]**	Improvised explosive	Solid	3.58	12.3	1500 (20 °C)	170	210	NO_2_
**Urea nitrate (UN) [24]**	Improvised explosive	Solid	~3.7 (NO_3_^−^)	0.009	150	163	unknown	NO_2_

**Table 2:** Electron affinity of NO_2_ relative to other molecular constituents of ambient air [25,26].

Gas	Name	Electron Affinity (eV)
**N_2_**	Nitrogen	−0.72
**O_2_**	Oxygen	0.448
**H_2_O**	Water vapor	negative
**CO_2_**	Carbon dioxide	−0.6
**H_2,_ HC**	Hydrogen, most hydrocarbons	≤0
**NO_2_**	Nitrogen dioxide	2.273
**O_3_**	Ozone	2.103

The electron affinity *EA*, in general, is defined as the amount of energy that is released when an electron is added to a neutral atom or a molecule to form a negative ion [27]:

X + e^−^ → X^−^ (1)

Reference to Table 2 reveals that the electron affinity of most air constituents is zero or negative, *i.e.*, energy is required to form negative molecular ions by means of electron capture. The most abundant molecule with a positive *EA* in ambient air is oxygen (O_2_; *EA* ~0.5 eV). In case free electrons are available, O_2_^−^ ions will therefore spontaneously form in clean ambient air:

O_2_ + e^−^ → O_2_^−^ (2)

In case ambient air becomes additionally contaminated with NO_2_ (*EA* ~2.5 eV) or O_3_ (*EA* ~2.1 eV), the electrons initially trapped on O_2_ molecules will spontaneously transfer in the course of gas-kinetic collisions from the O_2_^−^ ions to the NO_2_ or O_3_ ones:

O_2_^−^ + NO_2_ → NO_2_^−^ + O_2_ (3)

O_2_^−^ + O_3_ → O_3_^−^ + O_2_ (4)

**Figure 3:**
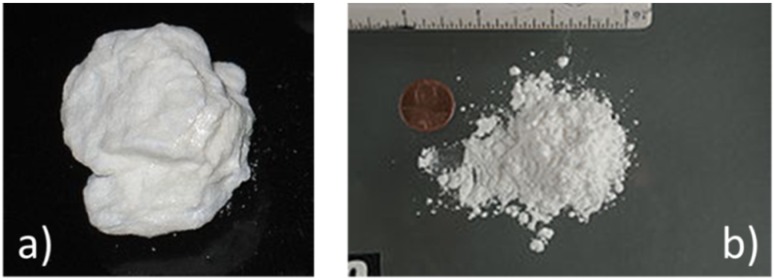
(**a**) Free-base form of cocaine; (**b**) salt-like cocaine hydrochloride.

This tendency of electron transfer to the highest electron-affinity molecules is called atmospheric pressure chemical ionization (APCI) [17,28]. As the vast variety of explosives either contains NO_2_ or NO_3_^−^ side groups on their hydrocarbon backbones (Figure 3), these threat materials also exhibit high electron affinities. Theoretical estimates range from about 1 up to 2.5 eV (Table 1). Once vaporized, explosives molecules can therefore easily take up negative charge from O_2_^−^ ions, thus forming negatively-charged analyte ions, which can then be analyzed with regard to their ion mass. This latter process is extensively used in the IMS detection of explosives (Section 3). Last, but not least, high electron affinities are also retained in the solid state. When the explosives still abound in solid form, their high electron affinity makes it easy to deposit electrons on explosive solid particle residue and to guide the charged residue out of clouds of less electron-affinity background matter. This latter possibility is exploited in the electrostatic particle precipitators described in Section 4.3 and used to purify sampled solid matter before it is thermally converted into detectable vapors.

### 2.2. Illicit Drugs

Illicit drugs, like explosives, also abound in the form of low vapor pressure molecular solids. As such, they also cannot be directly detected using conventional forms of gas or vapor detectors. Another specific feature of illicit drugs is that these can abound in two distinctly different forms,* i.e.*, in free-base and in salt forms. In the first form, the drugs are insoluble in water, while they are water-soluble in the latter. Figure 3 shows samples of cocaine in these two forms. The salt form can be obtained by treating the material in its free-base form with strong acids, as for instance HCl. The data compiled in Table 3 further show that, similar to explosives, illicit drugs can be easily transformed into detectable vapors by mild heating. Melting and boiling points again are in the order of 200 °C.

**Table 3:** Molecular properties of illicit drugs; ephedrine is an easily available drug surrogate.

Name	Comment	Aggregate Form	Melting Point Free-Base/Salt (°C)	Boiling Point (°C)	Proton Affinity (eV)
**Ecstasy [29]**	Drug	solid	152/11 (flash point)	155	8 to 10
**Cocaine [30]**	Drug	solid	98/197 (decomposes)	187	8 to 10
**Heroine [31]**	Drug	solid	171/250 (flash point)	273	8 to 10
**Ephedrine [32]**	Drug	solid	40/220	225	8 to 10

**Figure 4:**
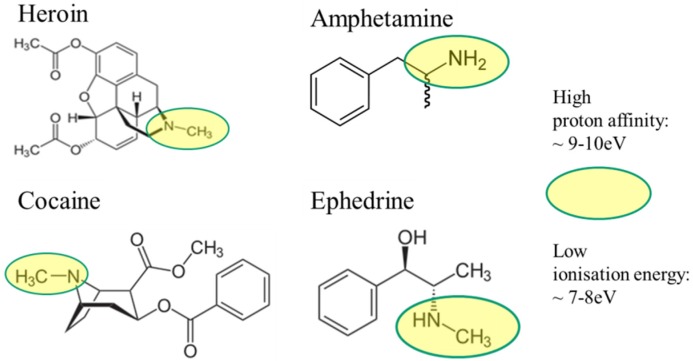
Molecular structure of illicit drugs. Ephedrine [32] is an easily available substitute for enabling efficient laboratory work. Amine functional groups are highlighted in yellow.

Once vaporized, the drugs can again be detected and distinguished against interfering substances on account of their specific molecular properties. Like explosives, illicit drugs feature a hydrocarbon skeleton and various functional side groups attached to this skeleton. The most important kind of side group in illicit drugs is amine functional groups (yellow ovals in Figure 4). The nitrogen lone pair electrons in these groups contain extra electronic charge, which makes them proton-attracting. In short: illicit drugs exhibit the important property of gas phase basicity and, thus, the ability to form positive ions by extracting protons from their chemical environments.

Unlike positive electron affinity, gas phase basicity is a fairly common molecular feature. Whenever available, all kinds of molecules tend to acquire protons from their chemical environments and, thus, will form positive ions. The ease with which this occurs depends on the value of the proton affinity *PA* of a molecule M. As in the case of electron affinity, *PA* is defined as the energy released upon performing the reaction [33]:

M + H^+^ → MH^+^ (5)

**Figure 5:**
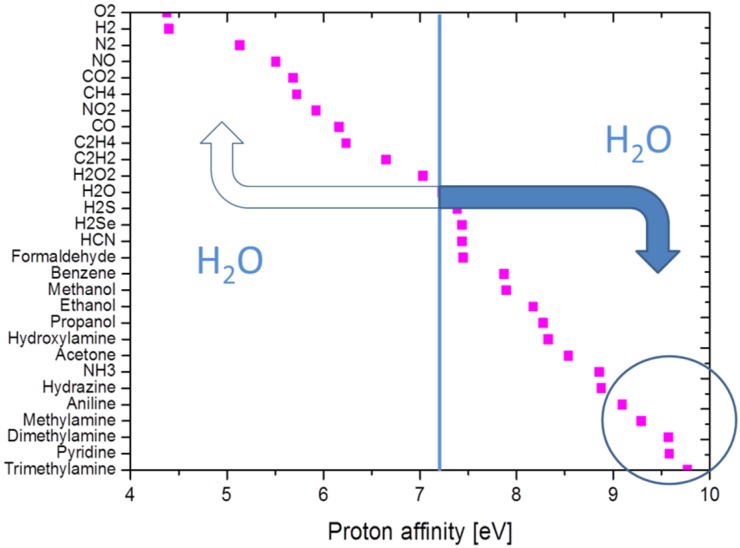
Proton affinity of selected molecules. The blue line highlights the proton affinity of water, *i.e.*, the energy released in the gas phase reaction H_2_O + H^+^ → H_3_O^+^. Starting from H_3_O^+^ ions, protons will be transferred to higher proton-affinity molecules (filled arrow) in the course of gas-kinetic collisions, while a transfer to lower proton-affinity ones is extremely unlikely (empty arrow).

Figure 5 lists tabulated values of the proton affinity [20,33], showing that molecules with amine functional groups are exceptional in the sense that these exhibit the highest proton affinities of all. In practice, this means that if gases with a different proton affinity are admixed to the ambient air, protons will be exchanged during gas-kinetic collisions until the protons have settled down on those molecules with the highest proton affinities. This latter process of proton transfer is another form of APCI, which is used in the IMS technology to detect illicit drugs.

## 3. Security Screening: State of the Art (IMS)

The standard way of detecting the above kinds of target materials is using ion mobility spectroscopy (IMS). In security screening, IMS is considered the “gold standard” against which any other kind of detector is to be compared and to be evaluated. IMS is a mature technology, which is widely applied in the security realm. An excellent introduction to this technique is given in the textbook of Eiceman [17].

**Figure 6:**
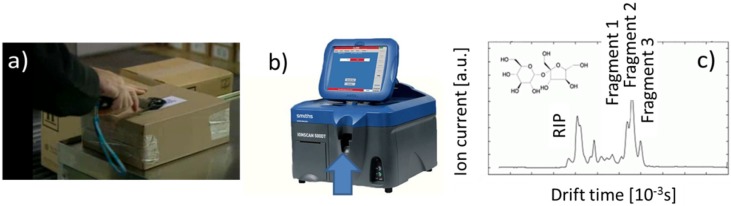
(**a**) Collecting trace particle residue from a suspect item using the IonScan 500 sampler “spoon”; (**b**) IonScan 500 ion mobility spectrometer (IMS) with the insertion port for the sampler spoon (arrow) [34]; (**c**) example of ion drift spectrum.

For the purpose of the present paper, it is important to understand how this technology works and how it is applied in typical security screening scenarios. The target of interest in security screening is usually an item that may contain either explosives or illicit drugs. Upon filling drugs or explosives into these items, their outside surfaces normally become contaminated with traces of these target substances. These traces usually consist of particle residue with diameters in the size of micrometers or below. These contaminations therefore are not normally visible to the unaided eye, but are very sticky and, therefore, very useful for examination. In order to collect such traces of target material, a so-called swab, essentially a piece of porous cloth, is moved across the suspect surface with the help of a sampling spoon (Figure 6a). This spoon is spring-loaded to exert a constant pressure on the searched surface. After wiping the suspect surface, the swap usually has taken up a considerable number of microscopically small target particles. With this particle load, the swap is inserted into the insertion port of an IMS (Figure 6b). Once inserted, a pre-heated metal rod is pushed up against the bottom-side of the swab, and the trace impurity materials trapped within this swab are vaporized into the ionization region of the IMS. There, either positive (illicit drugs) or negative (explosives) substance ions are generated, which are subsequently analyzed for their molecular masses. Overall, an IMS performs as a time-of-flight mass spectrometer working at full ambient pressure. Figure 6b shows an IonScan500 instrument (Smiths Detection, Hertfordshire, UK), a type of IMS that is widely used in international airports [34]. Its attractive feature is that it contains both a positive and a negative ion drift tube within the same instrument. Due to this versatility, it allows both explosives and illicit drugs to be detected within one piece of hardware. Figure 6c shows an example of an ion drift spectrum obtained after ionizing the molecules shown in the inset of this same figure. Table 4 summarizes main features of state-of-the-art instruments. Whereas the IonScan 500 uses radioactive ionization, more recent developments in the IMS field aim to replace the radioactive ionization and, thus, intend to make IMS instruments more easily usable. Alternatives to the widely-employed radioactive ionization are photo ionization [35], two-photon laser ionization [36] and corona ionization [37].

**Table 4:** Properties of state-of-the-art IMS instruments.

**Detectable substances**	Explosives, illicit drugs, chemical warfare agents (CWA), toxic industrial compounds (TICs)
**Detection limits**	Solids: 0.1 nanogram; gases, vapors (ppb)
**Speed of response**	Identification of sampled analyte within tens of seconds after flash evaporation
**Time required for sampling**	Dependent on suspect object and inspector
**Volume/weight**	Several liters/several kg
**Price**	~40 to 50 thousand €
**Downsides**	Use of radioactive ionization only allowed by specially-trained personal in approved locations.

**Figure 7:**
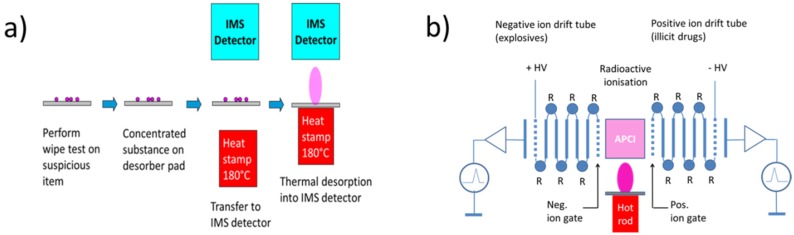
(**a**) Process of particle sampling and solid-vapor conversion for IMS detection; (**b**) internal architecture of an IMS, featuring an ionization region (magenta) and two ion drift tubes (blue) with associated detector circuits for positive (Pos.) and negative (Neg.) ions, respectively.

Working towards lower cost alternatives of IMS instruments, it is important to understand the key success criteria that have made the IMS technology so successful. The first success factor is the process of particle sampling and solid-vapor conversion, which is illustrated in a more schematic way in Figure 7a. Collecting the target substances in the form of solid particle residue instead of the extremely dilute vapors emerging from such residue opens up a chance to collect small amounts of target substance in concentrated form within a short time. Thermal solid-vapor conversion of the collected substance, on the other hand, produces short and relatively intense vapor flashes in response to an external trigger event that had been purposely generated by the operator. Sharply rising sensor signals in response to an external trigger event effectively allow any form of sensor drift to be combatted. Very obviously, this pre-detection process of particle sampling and thermal solid-vapor conversion is not limited to IMS, but a possible enhancement for any form of alternative backend vapor detector.

**Figure 8:**
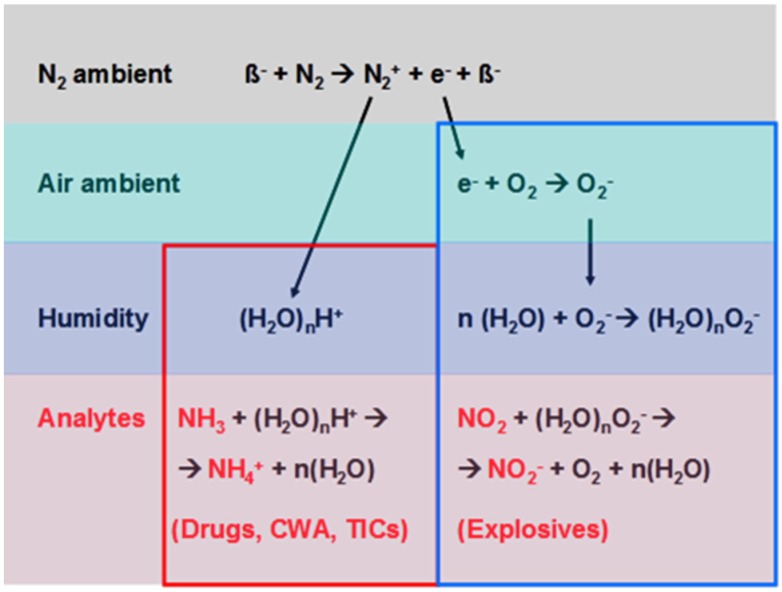
(Top panel) Radioactive ionization of N_2_ molecules by high-energy electrons (β-particles) leading to positive (hydrated protons) and negative (low-energy electrons) ionization products. In the follow-on atmospheric pressure chemical ionization (APCI) processes, the initial ionization products become attached to increasingly higher proton- or electron-affinity molecular species, thus forming positive (left) and negative (right) analyte ions, here represented by NH_3_ and NO_2_ ones. (CWA: chemical warfare agents; TICs: Toxic industrial compounds). For more details, see [17].

The second success factor of the currently-employed IMS technology is the generation of a sequence of atmospheric pressure chemical ionization events, which follow the initiating radioactive ionization. As shown in Figure 7b, APCI ionization in an IMS instrument is used to convert the generated vapor plumes into clouds of positive and negative ions, which can then be analyzed for their ion mass in the ion drift tubes left and right of the ionization region itself. Whereas in the IMS technology, the key interest is in the spectroscopic information contained in the drifting ion species, a large fraction of the explosives and illicit drug selectivity of IMS instruments derives from the gas-kinetic processes occurring in the ionization region itself. As illustrated in Figure 8, the atmospheric pressure chemical ionization (APCI) processes that immediately follow the initial radioactive ionization of the background air effectively transfer the initial ionization products (electrons and protons) onto those fractions of air constituents that feature either the highest electron or proton affinities. As the backend IMS detectors focus on the detection of ionized instead of neutral molecules, the predominance of APCI for high-electron and high-proton affinity species essentially removes any lower-affinity background from the competition of ionic detection in the backend IMS drift tubes. This latter form of selectivity enhancement by APCI processes is a major contributor to the high explosives and illicit drug selectivity of IMS instruments. As will be shown in more detail in Section 4.3, chains of APCI processes can also be triggered by corona discharges. In this form, the selectivity generating potential of APCI processes can also be employed at the pre-detection stage of particle sampling and, thus, be made productive for many other forms of non-IMS detector technologies.

## 4. Metal-Oxide-Based Security Sensor Systems

In this chapter, we build on the lessons learnt from IMS technology, namely that explosives and illicit drugs should be sampled in the form of solid particle residue and be evaporated into backend vapor detectors to produce short and relatively intense flashes of vapor. In the following, we report on experiments that had been carried out with the aim of assessing the feasibility of small and inexpensive MOX gas sensors to serve as backend vapor detectors [38,39,40,41,42]. As a first result, we show that combinations of thermal solid-vapor converters with MOX gas sensors are able to detect explosives particle residue (Section 4.1) and illicit drugs (Section 4.2) with sufficient sensitivity to live up to the demands of security threat detection. As a second result, we demonstrate in Section 4.3 that the intrinsic selectivity of such thermal converter-detector combinations can be considerably enhanced when the solid particle residue is acquired through electrostatic particle precipitators (ESP) [16]. In this way, the selectivity-generating power of APCI processes can be employed to separate the potential target particle residue from the huge background of analytically irrelevant dust.

### 4.1. Feasibility of MOX Gas Sensors in the Field of Explosives Detection

**Figure 9:**
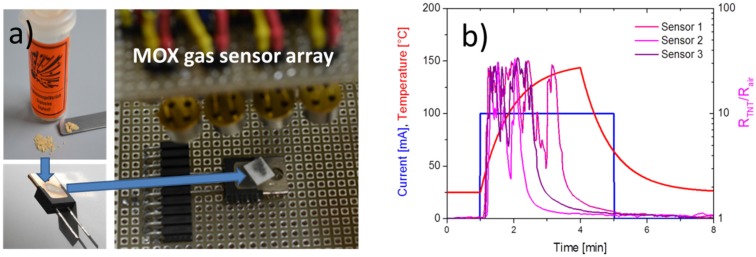
(**a**) Evaporation of TNT particles from an electrical heater into a MOX gas sensor array; (**b**) response of the sensors to the evaporating TNT particles (blue: current input into the heater element; red: resulting temperature ramp; colored curves: relative resistance response of the sensors to the resulting TNT vapor pulses.

The considerations in Section 2 have shown that sampled explosives particle residue melts and decomposes very easily, producing NO_2_- and NO*_x_*-containing molecular fragments. NO_2_, in turn, can be very easily and very sensitively detected using MOX gas sensors [43]. The principle feasibility of using MOX gas sensors to detect TNT is demonstrated in Figure 9.

In this experiment, small amounts of TNT were slowly evaporated from a commercial electrical heater element and detected in an array of eight identical MOX gas sensors (Figure 9a). As demonstrated in Figure 9b, the sensors respond with a series of sharp, oxidizing gas responses as the heater temperature is slowly ramped up. Due to the differing thermal contacts of the TNT grains to the heater surface, the individual grains deflagrate at random times, sending out flashes of oxidizing vapors towards one or several sensors in the array. Surprisingly, this fairly improvised sensing arrangement proved to be more efficient in providing a rapid and sensitive response towards TNT vapor flashes than many other well-designed measurement chamber configurations. We attribute this positive result to the fact that, in the arrangement of Figure 9a, the very sticky TNT vapors are directly driven towards the MOX gas sensors without giving them any chance to condense on any intermediate chamber walls.

**Figure 10:**
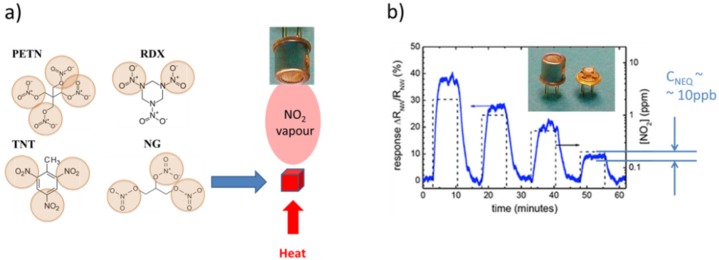

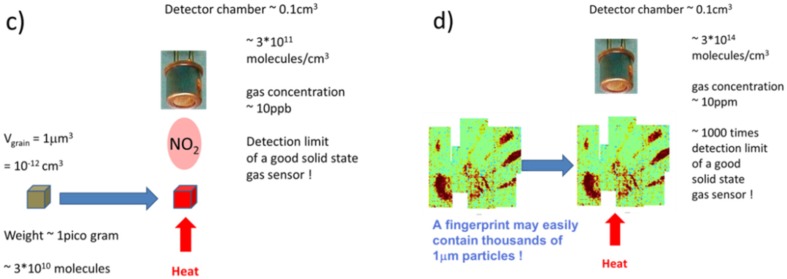
Use of MOX gas sensors for explosives detection: (**a**) explosives disintegration producing NO_2_ vapors; (**b**) NO_2_ sensitivity of a commercial NO_2_ sensor featuring a noise-equivalent resistive response of about 10 ppb; (**c**) an evaporation/disintegration of a 1-µm grain of explosive producing the noise-equivalent NO_2_ concentration; (**d**) thermal conversion of a hand/finger print of explosives producing an easily detectable NO_2_ concentration.

Considering the principle feasibility of MOX gas sensors for detecting explosives, the first question arises with regard to the ultimate sensitivity that may be attained. This question is treated in compact form in Figure 10. The main conclusion conveyed there is that NO_2_ vapors are liberated in the course of thermal evaporation and/or disintegration events of military high explosives (Figure 10a) and that the amounts of NO_2_ gases emerging from micrometer-sized grains should yield gas concentrations inside the tiny detector chambers to produce gas concentrations equivalent to the minimum detectable NO_2_ concentrations of commercially available MOX gas sensors (Figure 10b,c). The other conclusion is that a successful swab over a suspect object should yield enough material to produce a sensor output signal corresponding to 10^2−^ to 10^3−^times this minimum detectable amount (Figure 10d). Overall, this first assessment of MOX gas sensors suggests that masses of explosives particle residue well into the sub-nano-gram range should be detectable. A critical assumption in this whole argument is that all of the vapor molecules that result from the evaporation of the explosives particle residue can ultimately be collected inside the very small MOX detector volume. Considering the results of Figure 9 and the fact that deflagration of explosives particle residue tends to spread the vapors in random directions, it is important to keep the distance between the deflagrating materials and the detector small and to collect the dispersed vapors on heated metal surfaces, which prevent their re-condensation. In addition, airflows need to be applied to guide the released vapors into the tiny detector volume. Another important point to consider is that, in order to avoid fake signals, the sensors need to be heated using control circuits, which maintain the sensor operation temperature at a constant pre-set value independent of any changes in the airflow conditions at the sensor surface [44]. The key technical challenges, therefore, relate more to the design of efficient thermal converter-detector combinations than to the actual improvement of currently-available detector materials themselves.

**Figure 11:**
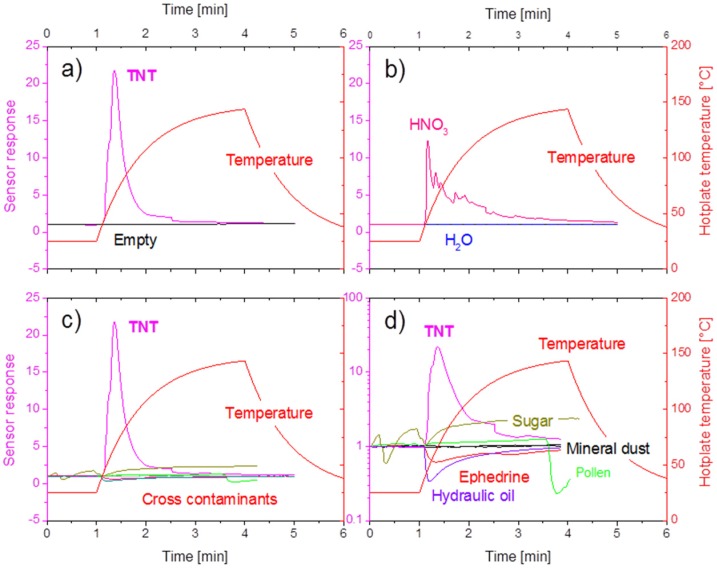
(**a**) Response of a single NO_2_ MOX sensor to an empty (black) and a TNT-loaded hotplate (magenta) when the hotplate temperature (red) is ramped up; (**b**) control experiment in which pure water (blue) and nitrous acid (HNO_3_) are evaporated; (**c**) control experiment in which many kinds of background particle (liquid) residue were evaporated; (**d**) same data as in (**c**), but plotted on a logarithmic scale.

A second key issue in the MOX detection of explosives is the problem of cross-sensitivity. This latter problem is addressed in Figure 11. In the experiments reported there, the same experimental equipment has been used as in the TNT evaporation tests above, but now, a large number of potential cross-interfering substances had been evaporated into the detector array. For the sake of comparison, Figure 11a repeats the variation of the sensor response, *i.e.*, the resistance ratio *R*_gas_/*R*_air_, as a small grain of TNT is evaporated from the hotplate surface. As expected, a strong oxidizing sensor signal is observed that fades away after the TNT had been completely evaporated. Similar responses are obtained upon evaporating other kinds of explosives. The black line, for comparison, shows the extremely small response when a temperature ramp is applied to an empty heater element. A simple, but potentially important interfering substance is water, as it can reside on all kinds of particle residues that may be collected on a swab. In this context, Figure 11b shows that an almost zero response is obtained when de-ionized water is evaporated. This situation, however, changes dramatically when a drop of HNO_3_ is evaporated. As HNO_3_ releases NO_2_ upon evaporation, a strong oxidizing gas response is observed. Figure 11c,d repeats the TNT sensor signal already reported in Figure 11a and compares it to those responses that were generated when the hotplate was loaded with particles that are likely to co-exist on suspect surfaces and that would therefore produce background signals. The particle loads considered came out of the three groups of mineral dust (sand grains, TiO_2_ nanoparticles), bio-contamination (pollen) and various organic backgrounds (oil, sugar and ephedrine). All of these background materials produce comparatively small reducing gas responses when evaporated in similar quantities. Overall, these cross-sensitivity tests show that MOX gas sensors respond more sensitively to substances that produce NO_2_ vapors upon evaporation as opposed to many reducing background materials, which do not. The observed predominance of oxidizing over reducing gas sensors signals, however, only amounts to a factor of 10 to 100, *i.e.*, to a ratio that is likely to be too small when a swab across a suspect surface has produced very little explosives residue, but a large quantity of reducing background stuff. As the small explosives signals may then become buried in the background of dominating reducing sensor signals, additional selectivity needs to be provided somewhere else in the detection chain.

**Figure 12:**
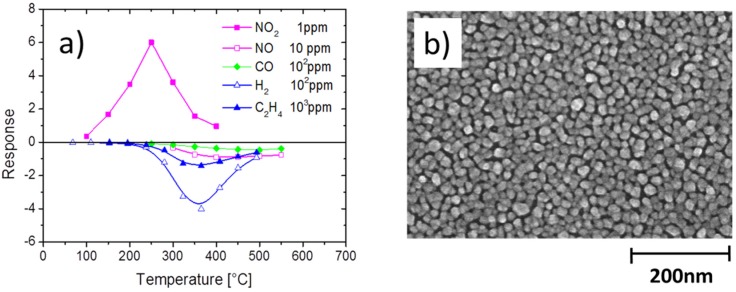
(**a**) Relative resistance change of a nano-granular SnO_2_ layer in response to oxidizing NO_2_ and a range of reducing background gases. A dominating NO_2_ response at low sensor operation conditions occurs due to the reactive nature of NO_2_ and its thermal instability at higher temperatures (NO_2_ → NO + ½ O_2_); (**b**) nano-morphology of the SnO_2_ sensing layer.

In the field of MOX gas sensors, the most straight-forward approach towards gaining higher selectivity is using arrays of similar sensors with different cross-sensitivity profiles and applying pattern recognition algorithms to the array output [45]. In order to motivate this approach, we present in Figure 12 gas sensitivity characteristics that have been measured on one single MOX gas sensor. From this example, it is apparent that different cross-sensitivity characteristics can be attained by operating identical sensors at different sensor operation temperatures. This kind of selectivity enhancement has been successfully used by Holl, *et al.* [46] to analyze off-gases that had been produced by flash-evaporating a range of explosives and background materials from micro-machined hotplate heaters. These authors very convincingly showed that all kinds of explosives can be safely distinguished from non-explosive background materials and that, moreover, different kinds of explosives materials can be distinguished from each other by performing principle component analysis on the sensor signals.

**Figure 13:**
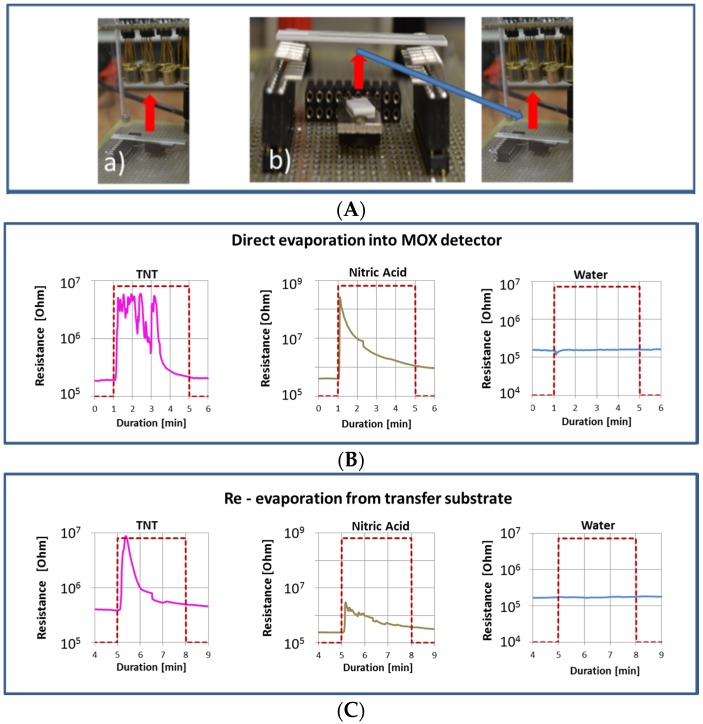
(**A**) One-step and two-step thermal solid vapor conversion processes; (**B**) sensor responses to TNT, HNO_3_ and water upon first evaporation; (**C**) sensor responses to TNT, HNO_3_ and water upon re-evaporation from the transfer substrate.

Tough problems are posed by interfering substances that do produce NO_2_ vapors upon flash evaporation and that therefore are likely to produce false positive alarms. In alleviating such problems, another approach to higher selectivity can be made at the pre-detection step of solid-vapor conversion. During this process, explosives and interfering compounds can be distinguished on account of their different stickiness on solid surfaces. This possibility is illustrated in Figure 13, where one-step and two-step thermal conversion processes are compared. As demonstrated there, TNT and HNO_3_, which both produce strong oxidizing gas signals upon first evaporation, behave very differently when re-evaporated from a transfer substrate. Due to their much lower stickiness, HNO_3_ and H_2_O get almost eliminated in the transfer process, while the TNT is fully recovered.

### 4.2. Feasibility of MOX Gas Sensors in the Field of Illicit Drug Detection

Similar to explosives, illicit drugs consist of hydrocarbon backbones with attached amine functional groups. Upon evaporation and fragmentation, a range of hydrocarbons and amines are produced (Figure 4). Unlike the explosives case, all fragments are reducing gas species. Considering the huge number of hydrocarbons, which could abound on suspect surfaces and which could be produced in an evaporation event, selectivity is very hard to attain using MOX gas sensors with a conventional RES readout.

**Figure 14:**
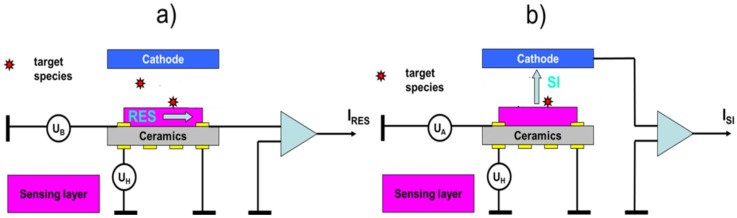
(**a**) MOX gas sensor operated in the conventional resistive (RES) and (**b**) in the innovative surface ionization (SI) readout mode. In the RES mode, gas adsorption is monitored via changes in the in-plane resistivity of the MOX sensing layers. The cathode layer is not normally present, but may be used to modify the gas adsorption via the electro-adsorption effect [52]. In the SI readout mode changes in the gas adsorption are monitored by observing flows of positive ions crossing the thin air gap (*d*_air_ ~ 0.1–1 mm) in between the heated MOX layer and the negatively-biased counter electrode.

Interesting options, however, open up in the case MOX gas sensors with surface ionization (SI) readout are used. This innovative kind of readout is illustrated in Figure 14 and compared there with the conventionally used resistive (RES) readout mode. As described in more detail in our recent papers [47,48,49,50,51], surface ionization can be observed when molecules become absorbed at heated solid surfaces and when a valence electron is transferred from the adsorbate molecule to the lowest energy unoccupied states within the adsorbent solid. In this way, positive ions are created, which can be extracted into free space by means of a negatively-biased counter electrode.

**Figure 15:**
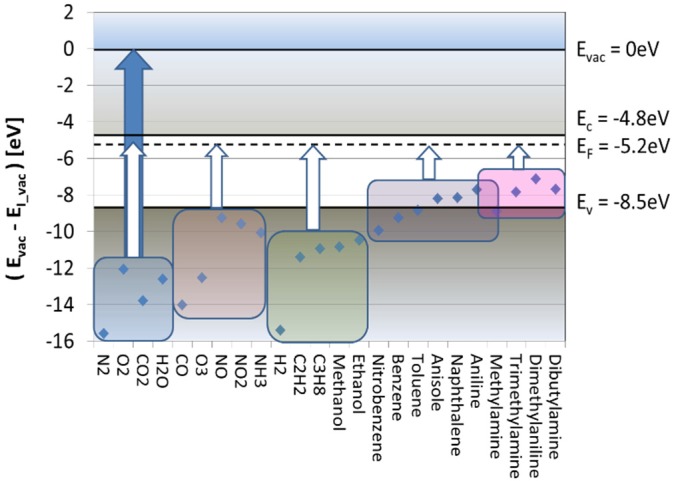
Free-space ionization energies $E_{I\_vac}$ of selected molecules [20] in relation to the vacuum energy $E_{vac}$ and the conduction and valence band edges and the Fermi energy in SnO_2_. Blue and white arrows: free-space and first-order surface ionization energies. Colored rectangles denote different groups of analytes.

In contrast to the conventional RES detection mode, which measures the oxidation or reducing power of an analyte, the SI mode measures its ease of ionization. Whereas in free-space ionization, energies between 7 and 16 eV are needed to raise a valence electron from an analyte molecule to an unbound vacuum state, surface ionization only requires an electron transfer from an adsorbed analyte to the lowest-lying unoccupied electron state inside the adsorbent solid. As demonstrated in Figure 15, no less than 5.2 eV can be saved if an analyte is first adsorbed on a SnO_2_ surface and then ionized by the transfer of a valence electron to the semiconductor Fermi energy. In this way, the surface ionization energy of analytes with very low free-space ionization energies can be reduced to something in the order of 2 eV, *i.e.*, to an amount that can easily be withdrawn from the thermal reservoir of a heated emitter material. A key advantage of the SI process is that the probability of thermal ionization scales in an Arrhenius-type manner with the ionization energy. In quantitative terms, the ionization current density $J_{ion}$ scales with the absolute temperature T and the analyte partial pressure $p_{A}$ as [47,48]:

(6) $$J_{ion}\;\sim \;p_{A}\;exp\left[-\frac{\left(\;E_{I\_vac}-\;E_{F}\right)\;+\;E_{ads}}{k_{B}T}\right]$$

In this equation, $E_{F}$ is the Fermi energy inside the adsorbent solid, $E_{I\_vac}$ the free-space ionisation energy of the adsorbate and $E_{ads}$ its binding energy on the emitter surface. Due to this exponential cut-off criterion, all stable air constituents, such as N_2_, O_2_, CO_2_ and H_2_O, remain completely undetectable. The lack of H_2_O sensitivity, in particular, is a significant advantage with regard to the conventional RES response, which is very sensitive to varying humidity backgrounds [38,49]. The relatively high ionization energies of air pollutants (CO, O_3_, NO, NO_2_) and of hydrogen and hydrocarbons also make these substances detectable only at very high emitter temperatures (>650 °C) [47,48]. Remaining candidates for relatively low-temperature ionization are aromatic hydrocarbons and amines. Aromatic hydrocarbons, however, are ruled out, as well as these feature exceptionally high adsorption energies $E_{ads}$ of up to 3 eV [48]. In practice, therefore, a high selectivity to amine-containing substances remains, which is a highly attractive feature if illicit drug detection is concerned.

**Figure 16:**
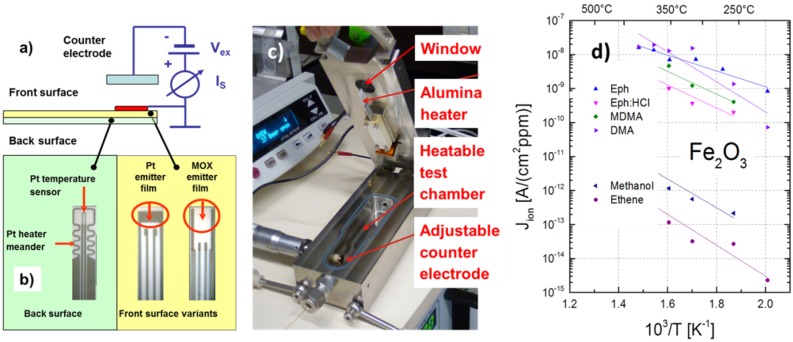
(**a**,**b**) Architecture of MOX sensors with SI readout; (**c**) measurement chamber for SI gas sensing tests. The active sensing area, determined by the size of the adjustable counter electrode, is ~10 mm^2^; (**d**) Ionization current density as a function of the temperature of a Fe_2_O_3_ emitter film. Amine-containing analytes (upper group) and non-amine-containing ones yield vastly different ion current densities.

**Figure 17:**
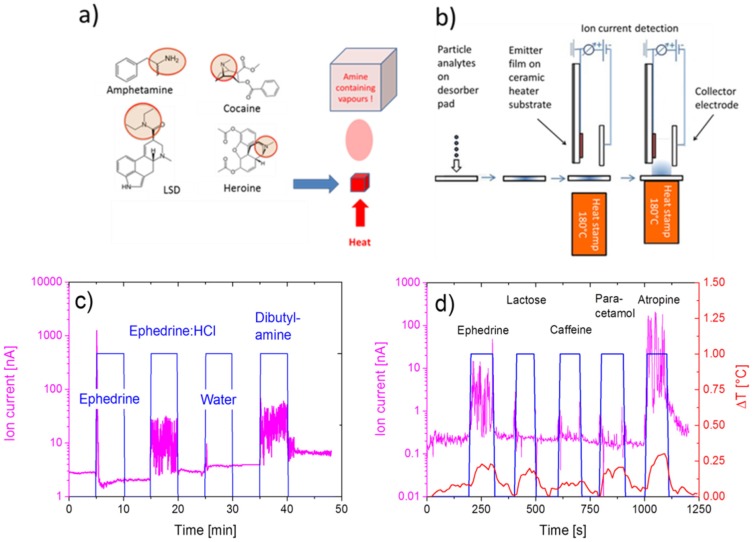
(**a**) Thermal disintegration of illicit drugs yielding hydrocarbon fragments with amine functional groups (circles); (**b**) process of collection and thermal desorption of analytes into the air gap of an SI detector; (**c**) SI current response to the evaporation of the drug substitutes ephedrine and ephedrine:HCl. For comparison, the responses to pure solvent water and to an organic decay product (dibutylamine) are shown [49]; (**d**) response to the evaporation of ephedrine and a range of cutting agents (lactose, caffeine, paracetamol, atropine), which usually contaminate street samples of illicit drugs [50,53,54,55,56]. The red line in (**d**) mirrors the temperature rise of the SI emitter film when the substance is evaporated into the air gap of the SI detector.

Figure 16 shows how this readout principle has been implemented in practice. The sensitive emitter films (Pt, SnO_2_, Fe_2_O_3_, CuO, *etc.*) were deposited on the front side of ceramic heater substrates with pre-deposited thick-film platinum (Pt) electrodes. On the backside, these same substrates carried Pt heater meanders and Pt thermometers (Figure 16a,b). During operation, the emitter films were kept at constant temperatures by feedback control circuits, which maintained constant, pre-set resistances of the Pt heater meanders. In order to enable SI readout, the heater substrates were mounted inside a heatable stainless steel chamber (Figure 16c). Within this chamber, the emitter films (active area ~13 mm^2^) were mounted opposite a micro-meter screw, which allowed a counter electrode to be moved into a well-controlled position above the emitter film (*d* ~ 1 mm). The resulting air gap could be controlled through a window in the top lid of the measurement chamber. Extraction potentials across this air gap typically amounted to about 1000 V. Gas access to the measurement chamber was provided by Swagelok inlet and outlet ports at both ends of the chamber. The whole chamber could be heated up to about 100 °C to reduce adsorption of the often very sticky analytes on the internal chamber walls. In the special case of the drug detection experiments reported below, the window in the top lid of the measurement chamber was removed, and the vapor flashes emerging from the solid-vapor converters sketched in Figure 17b were directly injected through the open window into the air gap of the SI detectors. Among those emitter materials that were tested so far, Fe_2_O_3_ proved to be the best performing one [49,50]. As shown in Figure 16d, Arrhenius-type ion current characteristics were obtained, and roughly four to five orders of magnitude higher currents were obtained from amine-containing substances as opposed to non-amine-containing ones.

With this equipment, drug sensing tests were performed. The method is schematically shown in Figure 17a,b, and the ion current responses observed upon evaporating drugs and potential interfering substances are shown in (c) and (d). The emitter film in both cases was a Fe_2_O_3_ film operated at a temperature of 400 °C. The blue rectangles in Figure 17c,d indicate the time spans during which the solid analytes were flash-evaporated into the air gaps of the Si-detector, and the magenta lines represent the SI currents generated. Overall, the data in Figure 17 show that all kinds of molecules with amine functional groups (ephedrine, ephedrine:HCl, dibutylamine, atropine) are detected, while others not featuring such functional groups go virtually undetected (solvent water, lactose, caffeine, paracetamol).

**Figure 18:**
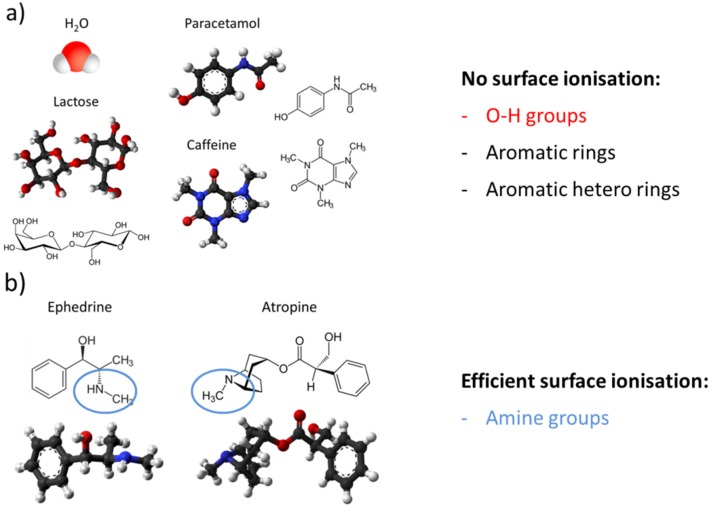
Molecular structures of the substances used in the evaporation tests reported in Figure 17. Color coding of atoms: carbon (black); hydrogen (grey); oxygen (red); nitrogen (blue): (**a**) substances not detectable by surface ionization, (**b**) substances well-detectable by surface ionization.

The importance of amine functional groups for enabling an SI response is further emphasized by the molecular structures of the molecules tested. Considering the fact that the substances displayed in Figure 18a could not be detected [52,53,54], it is apparent that molecules with O–H groups (H_2_O) will not be detected. This is not surprising considering the high free-space ionization energy of H_2_O (Figure 15). More surprising is the fact that aromatic rings, which feature low free-space ionization energies (Figure 15), also do not readily ionize. As mentioned above, the reason for this failure is a very tight adsorption of the aromatics on the emitter surface, which effectively increases their surface ionization energy [49]. A comparison of Figure 18a and Figure 18b shows that the successful ionization of ephedrine and atropine must be due to their amine functional groups (blue), rather than to their O–H (red) or aromatic (black) ones.

A clue to the exceptional role of amine functional groups is provided by the work of Fuji [57,58], who found, using mass spectroscopic analysis, that surface ionization mainly proceeds through three reaction channels. In decreasing order of probability, these are:

- Dissociative ionization: (M − H)^+^;
- Associative ionization: (M + H)^+^;
- Direct ionization: M^+^.

Calculation of the ionization energies for these three reaction pathways produced an energetic gap between amines, on the one hand, and non-amines, on the other hand. Ionization energies for dissociative surface ionization were found to be 6.0 eV for dimethylamine and 5.7 eV for trimethylamine, which is very close to the work function of SnO_2_ of about 5.2 eV. Ionization energies for all other substances, as for instance fluoro-benzene or toluene, are at least 2 eV higher. As the ionization current decreases exponentially as the ionization energy is increased, the enormous selectivity towards amines is explained.

**Figure 19:**
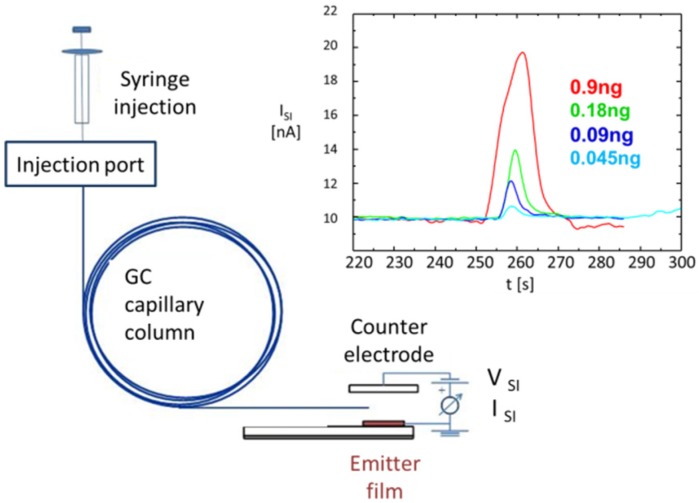
Injection of ephedrine into the air gap of an SI detector through a chromatographic column. The minimum detectable amount of ephedrine is less than 45 nanograms. Complete evaporation of this amount of ephedrine would yield a concentration of amine-containing molecules in the air gap in the high sub-ppm range.

Figure 19, finally, demonstrates the high sensitivity of SI detectors to amine-containing substances. In the experiment reported there, increasingly smaller concentrations of Eph:HCl were dissolved in methanol, and controlled quantities of solute were evaporated into the air gap of an SI detector through a chromatographic column. With this arrangement, the minimum detectable concentration of ephedrine was determined to be less than 45 nano-grams, a quantity that compares well to quantities detectable by IMS. This amount of mass sensitivity also agrees in its order of magnitude with direct gas sensing tests in which ephedrine and Eph:HCl were supplied through permeation tubes. These latter experiments yielded gas sensitivities in the order of several hundred ppb for ephedrine and tens of ppb for Eph:HCl [49].

### 4.3. Selective Collection of Particle Residue Using Electrostatic Precipitation (ESP)

The arguments presented above have shown that particle residue deriving from high explosives and illicit drugs can be detected with sufficient sensitivity once it is evaporated into appropriately configured MOX gas detectors. MOX gas sensors have also shown a certain degree of selectivity towards the key functional groups in both classes of target materials (NO*_x_* and amines, respectively). Overall, however, the selectivity of MOX gas sensors with RES or SI readouts cannot compete with the spectral resolution provided by IMS instruments. Whereas IMS detectors are able to distinguish different substances within a group of target materials, MOX detectors per se only exhibit more or less pronounced group selectivity. Below, we show that this group selectivity can be considerably enhanced, when the particle residue that is harvested from suspect objects is collected with the help of electrostatic particle precipitators (ESP) [16].

ESP samplers remove particle residue from a suspect surface by hydrodynamic forces to make it float in an air stream [59,60,61]. Once floating, the particle residue is forced through a corona discharge region where the particle residue may suffer gas-kinetic collisions with either O_2_^−^, OH^−^ or H_3_O^+^ ions, depending on the sign of the corona discharge and the humidity in the ambient air. In negative corona discharges, where O_2_^−^ and OH^−^ ions abound, explosives particles tend to capture electrons from these negative ions and, thus, become negatively charged. With negative charge accumulating on their surfaces, the explosives particles can be guided out of the sampling air stream onto a porous, electrically-conducting substrate, where they can be discharged and collected for later evaporation into a solid-state gas sensor. Similarly, in a positive corona discharge, where H_3_O^+^ ions abound, illicit drug residue tends to capture protons, which again allows it to be collected for later evaporation in a suitable vapor sensor. As these ESP processes exhibit obvious similarities to the APCI processes operating in IMS instruments, it is clear that the ESP processes will add selectivity to a security sensor system by either removing highly electron or highly proton-affinity substances from a large stream of analytically irrelevant background dust. As military high explosives and illicit drugs do exhibit such extremes in electron and proton affinities, highly purified samples of analytes will be collected on the porous collector electrode.

**Figure 20:**
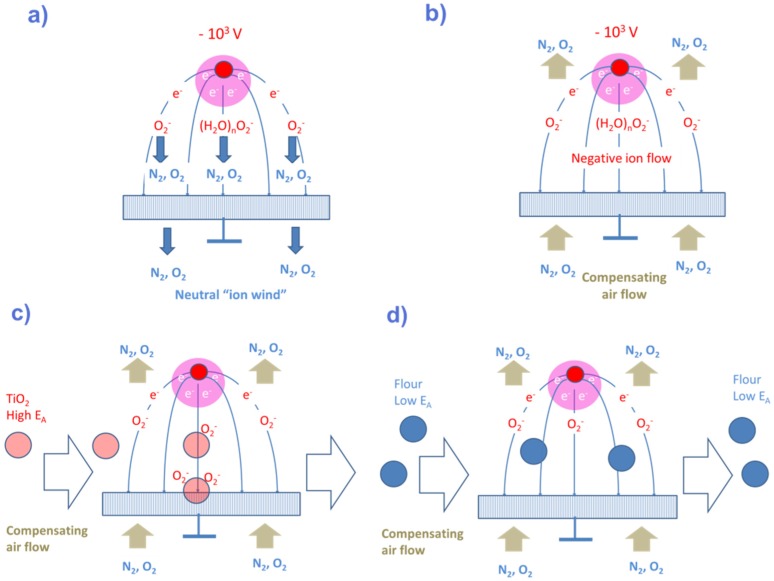
(**a**) Corona discharge arrangement generating a negative corona discharge around the sharply curved wire electrode and a stream of negative ions and a knock-on neutral ion wind moving towards a porous flat-plate collector electrode; (**b**) corona discharge arrangement with compensated ion wind; (**c**) corona discharge arrangement with compensated ion wind in the presence of a sampling air stream carrying particles with high electron affinity; (**d**) same arrangement as in (**c**), but with a sampling air stream carrying particles with a very low electron affinity.

The basic principles of ESP sampling are illustrated in schematic form in Figure 20a,b. Figure 20a shows a sharply curved metal electrode positioned in front of a porous, but essentially flat grounded metal grid. In practice, this curved electrode may take the form of a thin wire or a needle tip. Once a high negative potential is applied to such a curved electrode, a high electric field is generated in the immediate vicinity of the curved electrode, and a corona discharge is created. A corona discharge is initiated, once an air molecule (N_2_, O_2_) is ionized by an extraneous event (cosmic radiation, natural radioactivity, *etc.*). As the electrical field adjacent to the tip electrode is very high, the electrons and ions can pick up enough energy in between gas-kinetic collisions to impact-ionize further air molecules. In this way, cascades of positive ions and negative electrons are created. While the positive ions are rapidly extracted at the curved electrode, the electrons finally leave the high-field region and travel as a uniform flow of negative particles towards the flat-plate counter electrode. On their way towards the collector electrode, most electrons get stuck on O_2_ molecules, thus transforming them into O_2_^−^ ions. The O_2_^−^ ions, in turn, collide with neutral air molecules, thus pushing them into the same direction as the drifting O_2_^−^ ions. In this way, a neutral ion wind is formed, which tends to push all kinds of airborne particles, which cross the corona discharge region, towards the porous collector electrode, thereby immobilizing it there.

In order to make sure that only particles with a high electron affinity are immobilized on the collector grid, the ion wind needs to be overcompensated by a small opposing air stream, as shown in Figure 20b. In this latter arrangement, only the O_2_^−^ ions and any particles that had successfully acquired charge from these ions retain the ability of reaching the grounded collector electrode. Less electron-affine particles, in contrast, which failed to discharge O_2_^−^ ions, are swept away together with the sampling air stream. As the electrostatic forces scale with the specific charge, it is the fraction of very small, µm- and sub-µm-sized explosives particles that will ultimately become enriched on the sampling grid. After the sampling air stream had been stopped, the collector grid can be moved beneath a vapor detector, and its particle load can be evaporated into the vapor detector by passing a heating current through the collector grid and by vaporizing the particle load there. This same principle of selective collection can also be extended to illicit drug particle collection. In this latter case, the polarity of the corona electrode needs to be reversed to positive polarity [62]. In this case, a unipolar stream of positive H_3_O^+^ ions is created that is crossing the sampling air stream. As the H_3_O^+^ ions are created from N_2_^+^ ions interacting with H_2_O molecules (Figure 8), some humidity in the sampling air stream is required. Alternatively, the humidity needs to be supplied from some internal reservoir.

**Figure 21:**
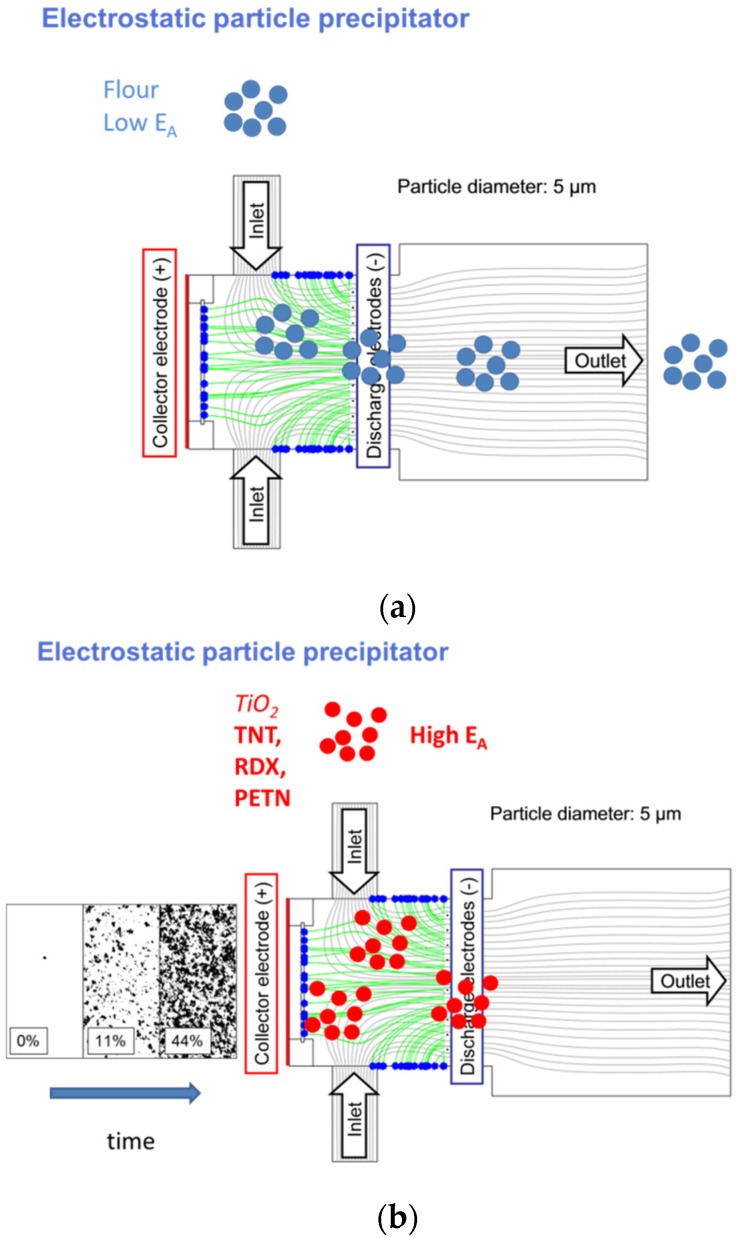
Sampling and precipitation of low (**a**) and high electron-affinity particles (**b**) using an ESP separator mounted on a hand-portable, battery-operated vacuum cleaner. While low *EA* particles (blue) follow the aerodynamic flow lines generated by the vacuum cleaner on paths connecting the inlet and outlet and thus become discarded, high *EA* ones (red) get negatively charged upon passing the corona wire array in the middle and get collected at the positively-charged collector electrode positioned opposite the air flow direction.

In order to demonstrate the selectivity of the ESP sampling process, a prototype instrument has been built as shown in Figure 21 [16]. In this instrument, a sampling air stream is generated by a small hand-portable vacuum cleaner. This vacuum cleaner, which is not shown in Figure 21, draws ambient air through two inlets towards the outlet port. On its way towards the outlet port, the air stream passes through an array of discharge wires. For explosives detection, these wires are strongly negatively biased (*V_bias_* ~ 5 kV), which causes corona discharges to form along each wire surface. Particles acquired by the sampling air stream and passing through this grid collide with O_2_^−^ ions and eventually get negatively charged. In case the sampled particles are small enough and have acquired enough charge, these particles attain the ability to move against the sampling air stream and to become collected at the positively-biased counter electrode on the far left-hand side. In order to illustrate the sensitivity of the ESP process with regard to the electron affinity of the sampled particles, Figure 21a,b displays two situations in which very low and very high electron-affinity particles were sampled. As model particles, we used flour (*EA* ~0 eV) for low *EA* particles and TiO_2_ nanoparticles (*EA* ~ 4 eV) for high *EA* ones. A comparison of both pictures shows that the low *EA* ones are transported to the outlet and become discarded, whereas the high *EA* ones become collected on the positively-biased collector electrode. The microscope images, shown in Figure 21b, show that the collector becomes increasingly covered with high *EA* particles as the sampling process is maintained for increasingly longer times.

**Figure 22:**
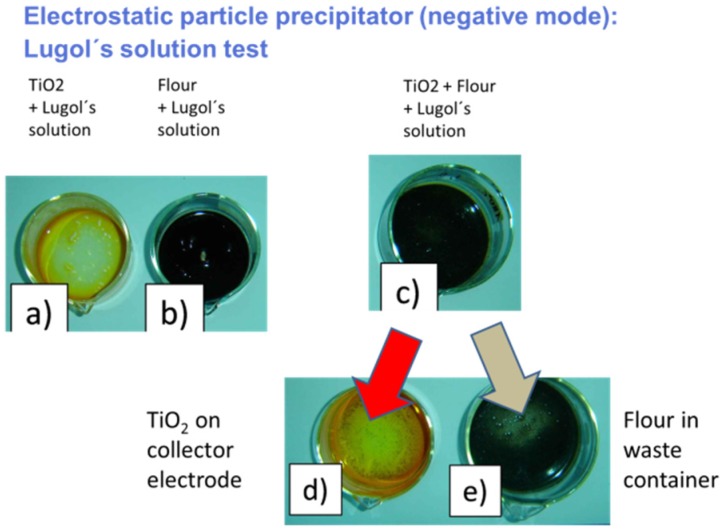
Lugol’s test applied to pure TiO_2_ (**a**) and to pure flour (**b**) demonstrating the presence of starch in (**b**); (**c**) Lugol’s test applied to a mixture of flour and TiO_2_; after passing this mix through the ESP separator, the mix is separated into almost pure fractions of TiO_2_ (**d**) and flour (**e**).

The ultimate proof of the selectivity of the ESP process is demonstrated when a mixture of flour and TiO_2_ particles is processed. In this case, we expect that a mixture, initially consisting of flour and TiO_2_ particles, is separated into its component powders, *i.e.*, TiO_2_ on the collector electrode and flour in the outlet container. That such a separation is indeed performed is shown in Figure 22. In this latter experiment, Lugol’s solution was added to the mixture offered to the inlet section of the ESP and to the two output powder fractions. As is well known, Lugol’s solution turns black when added to something that contains starch, while it stays transparent otherwise. Figure 22 shows that a mixture consisting of flour and TiO_2_ particles, which turns black in Lugol’s test, is separated into almost pure flour and pure TiO_2_ fractions after it has passed through an ESP separator.

**Figure 23:**
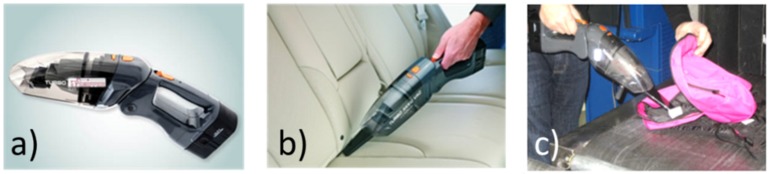
(**a**) ESP sampler system built into a commercial, battery-operated vacuum cleaner. Explosives particles and/or illicit drug residue were extracted from purposely contaminated environments (**b**,**c**) and placed onto cotton swabs inserted into the ESP sampler. Solid-vapor conversion and detection still needs to be performed by a separate stationary detector system.

Figure 23, finally, shows an advanced prototype of an ESP particle sampler as designed for the SNIFFER project [8]. This prototype builds on a commercial hand-portable vacuum cleaner, which was modified to include a custom-built ESP system and a commercial IMS collector swab. In order to improve the efficiency of the ESP process, the input air stream was forced through a curved channel to discard the fraction of larger particles via centrifugal forces before entering the corona discharge region. Due to this centrifugal filtering, the available ionic charge was more efficiently focused on the fraction of µm- and sub-µm particles, which are more easily deflectable by electrostatic forces and more easily convertible into detectable vapors. With this ESP sampler, extensive tests were performed with items purposely contaminated with traces of military high explosives and illicit drugs. After sampling, the analysis of the cotton swabs was carried out with the help of a commercial IMS instrument and, for comparison, with an innovative kind of vapor phase detector employing odor-binding protein-sensitive layers [63]. In all cases, contaminated items could be safely distinguished from non-contaminated ones, thus demonstrating the functionality of the ESP sampler device.

## 5. Achievements, Future Challenges and Outlook

### 5.1. Current Achievements

In our work, we have considered the current state of the art in security screening with a particular emphasis on the benchmark technology of IMS. Considering this successful technology, three key lessons have been learned, which are of general value beyond IMS technology:

- Explosives and illicit drugs abound on suspect surfaces in the form of low vapor pressure solid particles. Both kinds of target substances are best detected by collecting particle residue from the suspect surfaces and by thermally converting it into detectable vapors.
- As particles can be rapidly collected from suspect surfaces and rapidly evaporated, particle collection and thermal vapor conversion ensure speed, which is an important requirement in many security screening scenarios. Further, as solid particle residue contains high densities of target molecules, flash evaporation can produce relatively intense bursts of target vapor with peak concentrations well above the lower limit of detection (LOD) of the backend vapor detectors. Particle collection and flash evaporation are therefore also key enablers for high sensitivity.
- Once vaporized and subjected to showers of electrons and protons, cascades of APCI processes selectively place the electron and proton charges on explosive and illicit drug molecules, as these are normally those components in the evaporated gas mix, which feature the highest electron (explosives) or proton (drugs) affinities. In this way, the target materials can be electrostatically separated from lower affinity background matter. APCI processes therefore are key enablers for attaining high selectivity towards explosives and illicit drugs.

**Figure 24:**
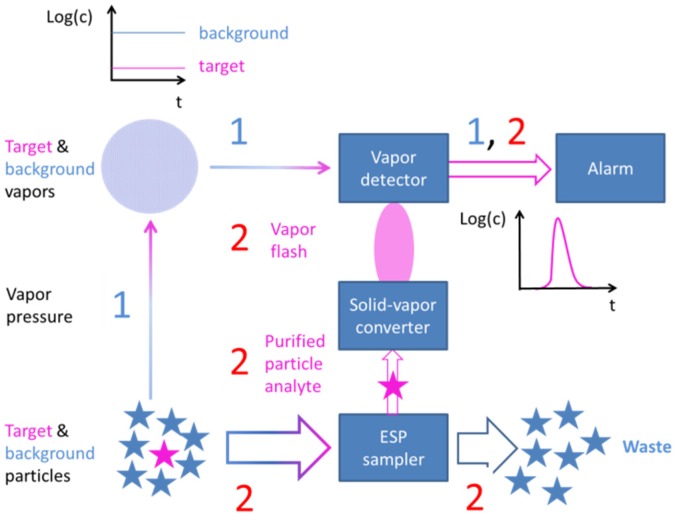
Detecting explosives and illicit drugs with the help of solid-state gas sensors. In Process Sequence 1, the small amounts of vapors emerging from the particle residue in the lower left corner are directly detected using a solid-state gas sensor. In Process Sequence 2, the solid particle residue is collected, purified, flash-evaporated and vapor-detected.

Keeping these lessons in mind, the sensor architecture of Figure 1 has been devised, which retains the IMS success criteria of particle collection, solid-vapor conversion and APCI sorting, replacing however IMS spectrometers as a backend vapor detection technology. Figure 24 illustrates the advantages of this sensor system approach (Process Sequence 2) and compares it to the direct gas sensing approach (Process Sequence 1), which focusses on the very low concentrations of gas molecules that emerge from the target particles on suspect surfaces. Following the IMS example, the target substance is collected in highly concentrated form in the form of solid particle residue and rapidly converted into detectable vapor, thus ensuring speed and sensitivity. Unlike the IMS example, the selectivity-generating power of APCI processes is not exploited at the final stage of backend vapor detection, but rather, in the frontend process of particle collection. In order to enable this, the swabbing of suspect surfaces had to be abandoned, and the particles had to be forced to float inside an air stream. In this way, the competition of aerodynamic and electrostatic forces on the floating particles could be used to separate high electron- or high proton-affinity particulate matter from the abundant low affinity background matter. In this way, ESP samplers do not simply dislodge particles from suspect surfaces, but rather provide samples of purified matter for the evaporation into the backend vapor detectors. Due to this selective sampling, intrinsically less selective solid-state gas sensors gain additional selectivity in their competition against IMS spectrometers.

Considering for the sake of definiteness sensor system architectures with readily available metal oxide gas sensors as backend vapor detectors, group selectivity towards the families of military high explosives and illicit drugs could be demonstrated. Explosives could be detected with lower limits of detection (LOD) in the nanogram range using commercial metal oxide gas sensors with a conventional RES readout. Illicit drugs could be detected with research types of MOX gas sensors with an innovative SI readout. With this latter kind of sensor, ephedrine could be detected with LODs in the range of several tens of picograms. Overall, our experiments have shown that integrated sensor systems featuring ESP particle samplers, thermal solid-vapor converters and backend vapor detectors can perform in the demanding fields of explosives and illicit drug detection. It is also obvious that our preliminary results leave plenty of room for improvement on all system levels, ranging from component, sensor integration to signal treatment and pattern recognition technologies. In the following, we briefly point out some of those potentials for further improvement.

### 5.2. Future Technology Enhancements

#### 5.2.1. Electrostatic Particle Precipitators and Solid-Vapor Converters

As described in Section 4.3 and visualized in Figure 23, ESP samplers do not simply dislodge particles from suspect surfaces [16,35,62], but they also sort them into fractions of analytically relevant and irrelevant fractions. As in the IMS detection of security threats, the sorting criteria are high electron or high proton affinity. The technology of electrostatic particle precipitation is, in fact, a very old one [60,61]. It is used on a large scale to remove particle loads from factory exhausts, and it has more recently been applied in electrostatic air cleaners. As far as we are aware, the use of ESP precipitators in the field of sensor technology is new [16,62]. For the purposes of the SNIFFER project [8], corona discharge electrodes and a porous, removable collector substrate were incorporated into a battery-operated, hand-portable vacuum cleaner to collect particle residue from suspect surfaces. Analysis of the collected matter was performed in a separate sensor unit, which contained a macroscopic entrance stage with a movable and permanently heated hot rod as in commercial IMS detector units. The sensor unit itself consisted of an array of mass-sensing diamond cantilevers onto which different kinds of odor-binding proteins (OBP) had been grafted [63].

Evolving from this state of the art into fully hand-portable detector units requires the backend functions of solid-vapor conversion and vapor detection to be integrated into the hand-portable ESP sampling unit. Arriving at this goal requires chip-sized units, which perform these backend functions. As discussed in Section 5.2.2, a variety of promising sensor technologies have already been developed that conform to such size constraints. The missing link in between macroscopic ESP precipitators and backend micro-sensor units is miniaturized hotplate heaters, which allow electrostatically-charged particulate matter to be discharged, collected, evaporated and efficiently injected into the backend vapor detector. In principle, such micro-electromechanical systems (MEMS) components are already available in the form of micro-heaters for MOX gas sensors, gas pre-concentrators, thermal mass flow meters and thermal infrared emitters [64,65]. These available components, however, cannot be directly re-employed as solid-vapor converters, as these have to fulfill a number of special requirements. As a first requirement, the micro-machined membranes need to exhibit sufficient roughness and/or macro-pores with sizes that allow micrometer and sub-micrometer particles to be electrically discharged and to be mechanically trapped until they are vaporized by thermal heating. Secondly, the generated vapors need to be captured on heatable entrance funnels, which effectively guide the analyte vapors into the backend vapor detectors. As military high explosives and illicit drugs are extremely sticky materials, the evaporated target materials will condense there, forming congruent thin films. Upon flash heating of these entrance ports, short, well-defined vapor flashes can be injected into the backend vapor detectors [66]. Constructing such MEMS systems is a demanding task, but clearly solvable using existing and widely-available technologies.

#### 5.2.2. Advanced Detector Technologies

**Figure 25:**
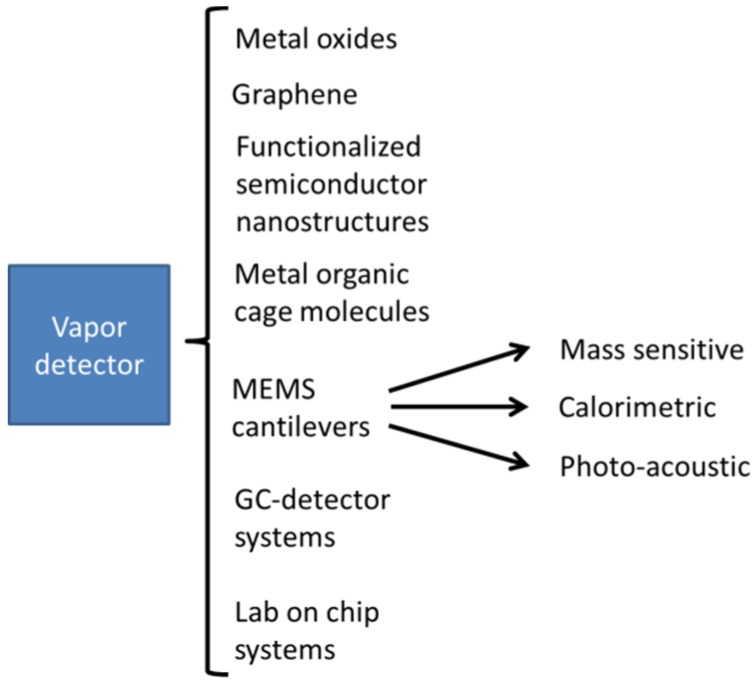
Families of chip-sized backend vapor detectors that form viable alternatives to the backend MOX gas sensors used in this work.

Reconsidering Figure 24, it is clear that the capability of rapidly collecting purified samples of solid particle residue and of converting it into short, detectable vapor flashes is not at all limited by the nature of the backend vapor detectors. Instead of the MOX gas sensors employed in the current work, a whole range of advanced and alternative sensor technologies can be exploited to perform the backend vapor detection task [13]. The key motivation in exploring such detector alternatives is achieving higher sensitivity and, perhaps more importantly, selectivity to individual threat substances, as it is possible in state-of-the-art IMS instruments. As illustrated in Figure 25, several families of sensors and chip-sized sensor systems are potentially available for this task.

In order to indicate the potential of such alternative sensor solutions, we only name a few examples. Concerning sensitivity, reports on graphene gas sensors have appeared which indicate that single-molecule sensitivity might be attained [67]. Such sensitivity would clearly outperform the sensitivity of existing IMS instruments. Other attempts at attaining high sensitivity involve nanostructures formed from mono-crystalline semiconductor materials with surfaces that had been modified to induce gas sensitivity [68,69,70,71,72,73,74,75,76]. In such nanostructures, gas sensitivity is induced by surface oxidation and/or by surface functionalization with specifically chosen organic ligands. Lower noise electrical readout is enabled by the superior transport properties of these single-crystalline materials, while interesting options for higher selectivity are enabled by the surface functionalization with organic molecules, metal-organic molecular cages, metal organic frameworks or odor binding proteins [63,77,78,79,80,81]. Another approach at attaining extreme sensitivity is employing extremely miniaturized MEMS cantilever devices, which can detect minute changes in mass loading or temperature changes [82,83,84]. Encouraging results have also been obtained using MEMS miniaturized gas chromatography detector systems [85,86,87] and multi-parameter detector systems [88,89,90]. In principle, all these detector approaches might form interesting backend sensor solutions in the integrated particle detection systems advocated in this work. Major unsolved issues are their coupling to the more macroscopic frontend devices of ESP precipitators and solid-vapor converters.
